# Plasminogen Deficiency: A Case Report and Review

**DOI:** 10.7759/cureus.45676

**Published:** 2023-09-21

**Authors:** Abdulrahman Nasiri, Marwa Nassar, Hazzaa Alzahrani

**Affiliations:** 1 Hematology, King Faisal Specialist Hospital & Research Centre, Riyadh, SAU; 2 Internal Medicine, Security Forces Hospital, Riyadh, SAU

**Keywords:** hepatic transplantation, therapy, replacement, hydrocephalus, conjunctivitis, ligneous, deficiency, plasminogen

## Abstract

Plasminogen deficiency, a rare disorder characterized by impaired fibrinolysis, frequently results in ligneous conjunctivitis. In this report, we report a case of a Saudi girl manifesting both conjunctivitis and hydrocephalus. Her initial symptoms at 1 month of age were recurring eye redness, which was inaccurately diagnosed as simple conjunctivitis. Surgical intervention for her ocular lesions revealed underlying membrane deposition. She later exhibited signs of increased intracranial pressure, resulting in a hydrocephalus diagnosis and subsequent surgery. Genetic analysis confirmed the presence of plasminogen deficiency. Clinical evaluations highlighted ligneous conjunctivitis, variations in visual acuity, and facial acne. Laboratory assessments demonstrated diminished plasminogen levels. The therapeutic approach encompassed plasminogen replacement, administered intravenously (1000 units, thrice weekly) and as eye drops, with the potential addition of fresh frozen plasma. Notably, this replacement therapy led to a significant reduction in hospital admissions and the severity of her conjunctivitis. Given the challenges in procuring consistent plasminogen supplies, the viability of hepatic transplantation is currently under investigation.

## Introduction

Plasminogen deficiency is a rare autosomal recessive disorder characterized by impaired fibrinolysis and fibrin accumulation. Ligneous conjunctivitis is a hallmark ocular manifestation of this deficiency. We present a case of plasminogen deficiency in a Saudi girl with ligneous conjunctivitis and occlusive hydrocephalus.

## Case presentation

Patient information

We report a 26-year-old female with a devastating history that started when she was one month old with recurrent attacks of redness and tearing in her bilateral eyes, for which she was diagnosed as having conjunctivitis. Both local and systemic treatments of antibiotics failed to improve the recurrences.

Following this inflammation, the family noted growing lesions on both eyes and then sought medical attention. During her evaluation, membrane deposition on the palpebral conjunctivae was noted, and she was kept under observation.

Thereafter, in her second month of life, she started to have projectile vomiting and abnormal posturing. She was diagnosed with occlusive hydrocephalus and had a ventriculoperitoneal shunt (VP) placement. One month later, the shunt became obstructed, leading to a repeat procedure for VP shunting.

The constellation of these symptoms dictated further evaluation, which included a comprehensive genetic history and laboratory evaluation.

The history revealed no consanguinity, no such presentation or other disorders in the family, and her basic laboratory tests were normal. The eye lesions were examined surgically for better evaluation, and lesions of variable sizes were found attached to the conjunctival surface of the lid of the right eye. The left eye examination revealed a substantial lesion on the inner eyelid, measuring approximately 1.2 mm x 6 mm in size.

These lesions were excised and sent for histopathological review. The lesions showed chronic inflammation, fibrinous material, granulation tissue, and fibrosis, consistent with ligneous conjunctivitis.

On further investigation, she was proven to be a homozygous carrier of plasminogen deficiency, which resulted in occlusive hydrocephalus as well as ligneous conjunctivitis.

The family members were screened and found to have heterozygous plasminogen deficiency.

Clinical findings

**Figure 1:**
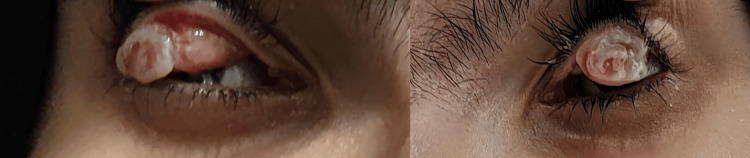
A yellowish-white pedunculated pseudomembrane, indicative of ligneous conjunctivitis on both eyes

**Figure 2:**
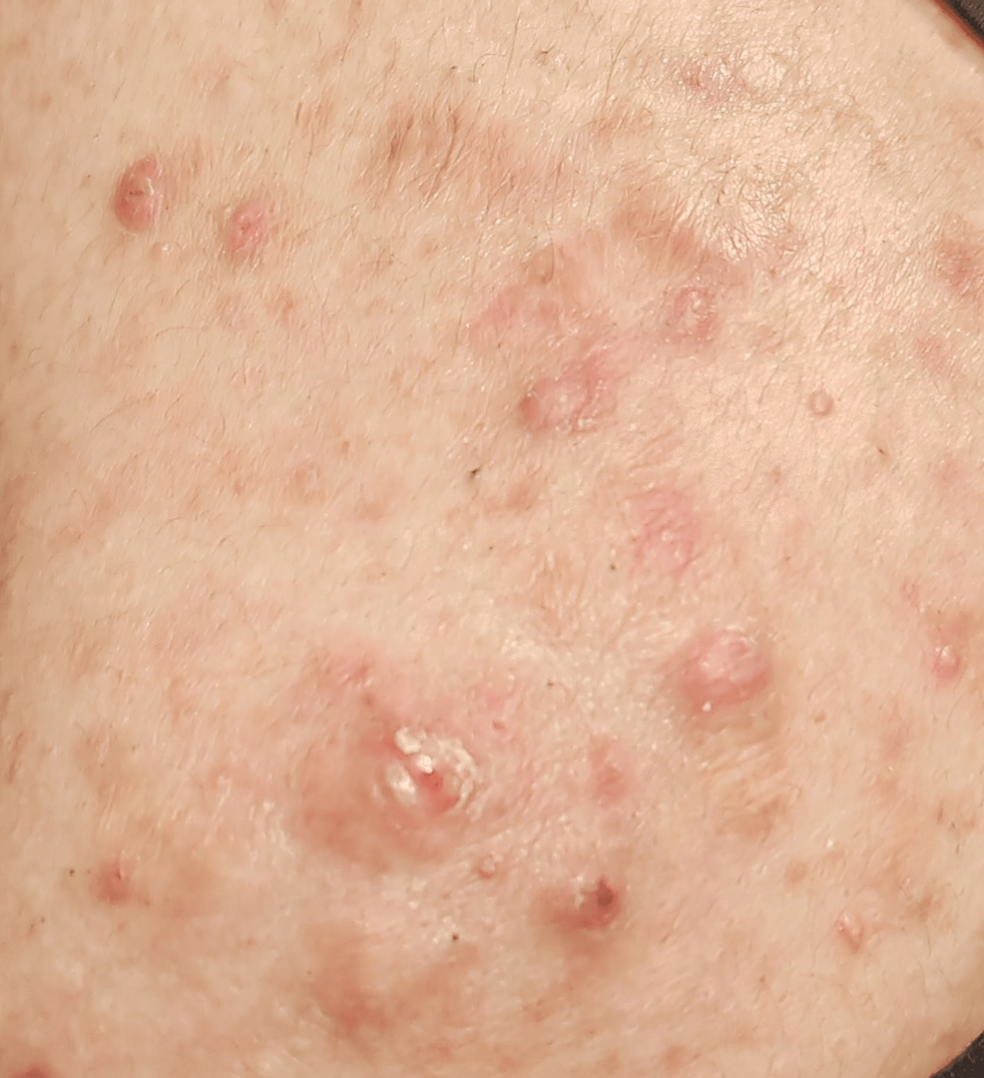
Nodulocystic acne on the back

The examination of the patient revealed a notable presence of a yellowish-white pedunculated pseudomembrane, indicative of ligneous conjunctivitis (Figure 1). Her visual acuity was measured at 20/25 in the right eye and 20/100 in the left eye. Furthermore, her skin exhibited nodulocystic acne on the back, accompanied by pustules across the face (Figure 2).

Diagnostic assessment

The complete blood count with differential showed a white blood count of 7.93X10^9^/L, hemoglobin of 13.5 g/L, and platelets of 321 X 10^3^ μl. Her prothrombin time ( PT) was 14.0 seconds and activated partial thromboplastin time (aPTT) was 38 seconds with an international normalized ratio (INR) of 1.2, and, notably, her plasminogen level was 20% (Table 1).

**Table 1:** Laboratory tests

Lab	Result	Normal value
Complete blood count		
WBC (10^9^/L)	7.93	4.5-13.5
RBC (10^12^/L)	4.98	3.8-6.5
HGB g/L	13.5	11.5-180
HCT %	42	36-48
MCV fl	85.1	77-98
MCHC g/L	318.0	310-360
PLT 10^3^ X μl	321	150-400
Coagulation profile		
PT (in seconds)	14.0	10.0-14.1
INR	1.2	0.86-1.2
APTT (in seconds)	38.0	24.6-40.1
Plasminogen level %	20	80-120
D- Dimer ug/ml	0.47	0.00-0.50

Therapeutic intervention

Following the identification of the deficiency, the patient was initiated on plasminogen replacement therapy by the team, administered intravenously (IV) using 50 vials, each vial containing 20 units, resulting in a total of 1000 units administered with IV three times per week. Additionally, plasminogen eye drops were introduced, leading to a decreased requirement for surgical excision. In situations where a shortage of plasminogen replacement arises, or its feasibility is in question, the patient would have been managed with fresh frozen plasma infusions (FFP).

Follow-up and outcomes

Following plasminogen replacement therapy, there was a dramatic decrease in her admissions and ligneous conjunctivitis formation.

Due to challenges posed by the availability, the cost of plasminogen replacement, and the side effects of FFP, which include allergic reactions, transfusion-related acute lung injury (TRALI), transfusion-associated circulatory overload (TACO), transmission of infections, volume overload, hemolysis, and febrile nonhemolytic reactions, significantly affecting the patient's quality of life, a consultation was initiated with the hepatic transplantation team. The feasibility of hepatic transplantation is currently under investigation at our center. Further publications may follow this article based on the outcomes of this investigation.

## Discussion

Plasminogen deficiency (PLG) is considered to be an ultra-rare, underdiagnosed autosomal recessive, multisystem disorder caused by mutations in the PLG gene resulting in two types, congenital type I plasminogen deficiency (hypoplasminogenemia) and congenital type II plasminogen deficiency (dysplasminogenemia) [1]. Its disease prevalence is estimated to be around 1.6 per million, and it exhibits a subtle female-to-male predominance [2].

PLG is primarily synthesized in the liver, circulates in plasma at a concentration of approximately 2 micromolars, and it is an integral part not only in hemostasis but also in wound healing, migration of cells, and even in embryogenesis [3,4].

**Figure 3:**
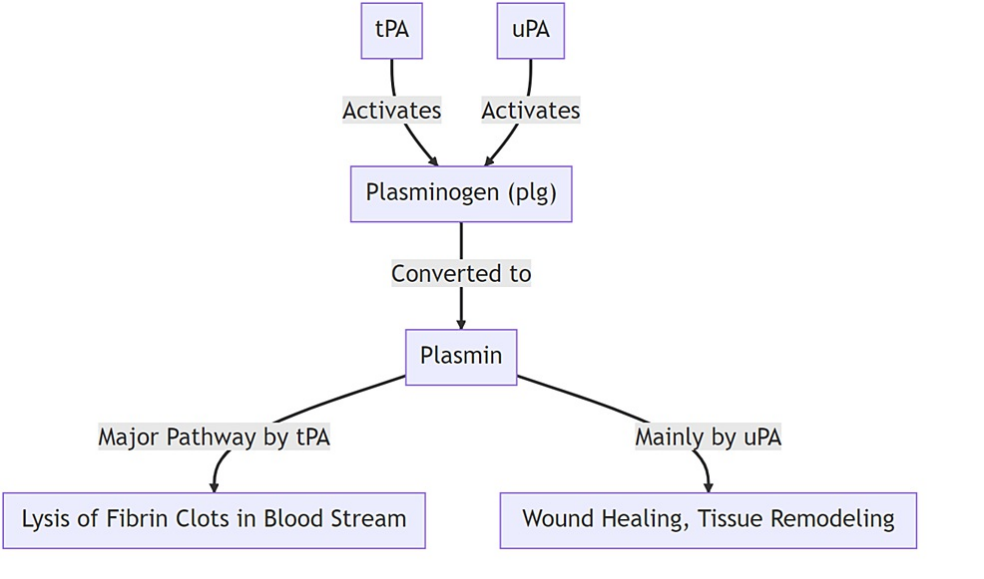
Plasminogen activation pathways

PLG activity is triggered by its conversion by tissue-plasminogen activator (tPA) and leads to the dissolution of the formed clots in the bloodstream (Figure 3). Furthermore, PLG activity can be triggered by urokinase-type plasminogen activator (uPA) and serve in wound healing and remodeling of the tissues [3,5].

The onset of disease exhibits chronological variability as a subset of patients manifest symptoms early in their lifespan and present with hydrocephalus [6]. Conversely, a subset of patients receives a diagnosis during their fifth decade of life [7]. Notably, type 2 patients typically remain asymptomatic and are identified primarily through family history and diagnostics labs [8].

The spectrum of manifestation is based on the fact that plasminogen deficiency leads to the accumulation of the fibrin-rich matrix in various parts of the body leading to the classical presentation of Ligneous conjunctivitis, where the patient presents with chronic tearing and redness following the presence of pseudomembranes of “wood-like” pedunculated white, yellow-white, or red masses on the conjunctiva. The same lesions can be found in the gingiva termed ligneous gingivitis, in the middle ear and tympanic membrane predisposing the affected individuals to otitis media and loss of hearing. The ligneous lesions can also be found in the respiratory tract leading to pneumonia and airway obstruction [7,9,10].

Ligneous cervicitis is another common place for the growth of the pseudomembranes among other structures of the female genital tract where they present with dysmenorrhea in addition to infertility due to growth on fallopian tubes, ovary, and endometrium [11]. The male genital tract is not spared from ligneous growth, and male infertility has been reported [12].

The skin can be affected by healing impairment and the presence of yellowish papules known as juvenile colloid milium, distinguished by clear papules found on sun-exposed regions [13].

The central nervous system is also involved in the deficiency of PLG, where a patient might present with congenital hydrocephalus [6]. A recent case report of a young male affected by cerebral infarction with no other clear cause was found apart from PLG deficiency [14].

As part of the hemostasis system and coagulation cascade, the loss of plasminogen activity has not been found to increase the risk of thrombosis in the affected patients, and the condition is not considered to be hypercoagulable [15].

Following history-taking with a focus on the presence of wound-healing abnormalities, family history of the same illness, and excluding acquired causes like tranexamic acid use, and physical examination looking for the ligneous, laboratory testing for PLG antigen (immunologic assay) and activity (chromogenic assay) is needed to confirm the diagnosis [16,17].

Genetic testing that discloses pathogenic variants in PLG can also provide diagnostic confirmation [9]. The treatment of PLG deficiency is by the restoration of the normal level by administering a human plasma-derived plasminogen; by replacing it, the body regains its fibrinolysis ability, and resolution of the formed ligneous lesions ensues in the affected parts with regaining of the normal function.

In 2021, a breakthrough in the management of PLG deficiency was represented by the approval of the Food and Drug Administration of Purified plasminogen derived from human plasma administered every two to four days at a dose of 6.6 mg/kg of body weight [18].

However, access to medication might prove to be difficult either because of pricing or availability, leading to utilizing other alternatives such as fresh frozen plasma. The exact amount of PLG in FFP is unknown and is associated with risks of infection, transfusion-related reactions, and volume overload [19].

In a recent case series from Italy, plasminogen concentrate eye drops led to resolution of the ligneous conjunctivitis [20]. Other less-optimal treatments include surgical excision, which is initially helpful; however, it results in pseudomembranes' regrowth. Oral estrogen-containing contraceptives appear to increase the level of PLG. Glucocorticoids, cyclosporine, and azathioprine were used with only limited benefit [21,22].

PLG deficiency prognosis varies, and it can significantly impact the quality of life of affected patients by causing loss of organ function, such as vision impairment, dental issues, and respiratory failure, in addition to potentially leading to organ failure.

## Conclusions

Plasminogen deficiency is an ultra-rare underdiagnosed disorder caused by mutations in the PLG gene, leading to conditions like congenital hydrocephalus and ligneous conjunctivitis. Less-optimal treatments have been used with limited benefit, while purified plasminogen is approved for treatment.

This case highlights the complexities of managing the disorder, with an emphasis on collaboration and innovative therapies. The patient's response to treatment and the consideration of hepatic transplantation illustrate avenues for improving her quality of life.

## References

[1] Mehta R, Shapiro AD (2008). Plasminogen deficiency. Haemophilia.

[2] Shapiro AD, Nakar C, Parker JM (2018). Plasminogen replacement therapy for the treatment of children and adults with congenital plasminogen deficiency. Blood.

[3] Schuster V, Hügle B, Tefs K (2007). Plasminogen deficiency. J Thromb Haemost.

[4] Edelberg JM, Enghild JJ, Pizzo SV, Gonzalez-Gronow M (1990). Neonatal plasminogen displays altered cell surface binding and activation kinetics. Correlation with increased glycosylation of the protein. J Clin Invest.

[5] Bugge TH, Flick MJ, Danton MJ (1996). Urokinase-type plasminogen activator is effective in fibrin clearance in the absence of its receptor or tissue-type plasminogen activator. Proc Natl Acad Sci USA.

[6] Karadag-Oncel E, Cengiz AB, Orhan D, Meral A (2015). A puzzling case: hypoplasminogenemia, ligneous conjunctivitis and hydrocephalus. Pediatr Hematol Oncol.

[7] Rodríguez-Ares MT, Abdulkader I, Blanco A, Touriño-Peralba R, Ruiz-Ponte C, Vega A, Cameselle-Teijeiro J (2007). Ligneous conjunctivitis: a clinicopathological, immunohistochemical, and genetic study including the treatment of two sisters with multiorgan involvement. Virchows Arch.

[8] Okamoto A, Sakata T, Mannami T (2003). Population-based distribution of plasminogen activity and estimated prevalence and relevance to thrombotic diseases of plasminogen deficiency in the Japanese: the Suita Study. J Thromb Haemost.

[9] Tefs K, Gueorguieva M, Klammt J (2006). Molecular and clinical spectrum of type I plasminogen deficiency: a series of 50 patients. Blood.

[10] Schuster V, Seregard S (2003). Ligneous conjunctivitis. Surv Ophthalmol.

[11] Pantanowitz L (2004). Ligneous cervicitis. BJOG.

[12] Altıner Ş, Klammt J, Bernhard MK, Schuster V, Karabulut HG (2017). Type I plasminogen deficiency with unexpected clinical aspects: Could be more than coexistence?. Cogent Med.

[13] Voicu C, Lisievici C, Coman C, Tebeica T (2019). Juvenile colloid milium: case report and literature review. Maedica (Bucur).

[14] Chen X, Zou M, Lu C (2023). Analysis of cerebral infarction caused by dysplasminogenemia in three pedigrees. Front Genet.

[15] Brandt JT (2002). Plasminogen and tissue-type plasminogen activator deficiency as risk factors for thromboembolic disease. Arch Pathol Lab Med.

[16] Diamond JP, Chandna A, Williams C, Easty DL, Scully C, Eveson J, Richards A (1991). Tranexamic acid-associated ligneous conjunctivitis with gingival and peritoneal lesions. Br J Ophthalmol.

[17] Klammt J, Kobelt L, Aktas D (2011). Identification of three novel plasminogen (PLG) gene mutations in a series of 23 patients with low PLG activity. Thromb Haemost.

[18] Kuehn BM (2021). First treatment for plasminogen deficiency is approved. JAMA.

[19] Kızılocak H, Ozdemir N, Dikme G (2018). Treatment of plasminogen deficiency patients with fresh frozen plasma. Pediatr Blood Cancer.

[20] Sartori MT, Bartuli A, Siboni SM (2023). Ligneous conjunctivitis and use of human plasminogen eyedrops: the Italian experience. Haemophilia.

[21] De Cock R, Ficker LA, Dart JG, Garner A, Wright P (1995). Topical heparin in the treatment of ligneous conjunctivitis. Ophthalmology.

[22] Teresa Sartori M, Saggiorato G, Pellati D (2003). Contraceptive pills induce an improvement in congenital hypoplasminogenemia in two unrelated patients with ligneous conjunctivitis. Thromb Haemost.

